# Comparing survey and multiple recruitment–mortality models to assess growth rates and population projections

**DOI:** 10.1002/ece3.5725

**Published:** 2019-10-22

**Authors:** William J. Severud, Glenn D. DelGiudice, Joseph K. Bump

**Affiliations:** ^1^ Department of Fisheries, Wildlife, and Conservation Biology University of Minnesota Saint Paul MN USA; ^2^ Forest Wildlife Populations and Research Group Minnesota Department of Natural Resources Forest Lake MN USA

**Keywords:** aerial survey, *Alces alces*, moose, population growth, recruitment–mortality Equation, survival

## Abstract

Estimation of population trends and demographic parameters is important to our understanding of fundamental ecology and species management, yet these data are often difficult to obtain without the use of data from population surveys or marking animals. The northeastern Minnesota moose (*Alces alces* Linnaeus, 1758) population declined 58% during 2006–2017, yet aerial surveys indicated stability during 2012–2017. In response to the decline, the Minnesota Department of Natural Resources (MNDNR) initiated studies of adult and calf survival to better understand cause‐specific mortality, calf recruitment, and factors influencing the population trajectory. We estimated population growth rate (*λ*) using adult survival and calf recruitment data from demographic studies and the recruitment–mortality (R‐M) Equation and compared these estimates to those calculated using data from aerial surveys. We then projected population dynamics 50 years using each resulting *λ* and used a stochastic model to project population dynamics 30 years using data from the MNDNR's studies. Calculations of *λ* derived from 2012 to 2017 survey data, and the R‐M Equation indicated growth (1.02 ± 0.16 [*SE*] and 1.01 ± 0.04, respectively). However, the stochastic model indicated a decline in the population over 30 years (*λ* = 0.91 ± 0.004; 2014–2044). The R‐M Equation has utility for estimating *λ*, and the supporting information from demographic collaring studies also helps to better address management questions. Furthermore, estimates of *λ* calculated using collaring data were more certain and reflective of current conditions. Long‐term monitoring using collars would better inform population performance predictions and demographic responses to environmental variability.

## Graphical abstract

Estimation of population trends and demographic parameters is important to understanding fundamental ecology and species management, yet these data are often difficult to obtain. We assessed growth rates and population projections of a declining moose population in Minnesota using aerial survey and multiple recruitment‐mortality models. Aerial survey results indicated that the moose population was stable during 2012–2017, and population modeling suggests that the population may have increased in the short‐term; however, over the long‐term, models made varying predictions about the direction of the population trajectory.

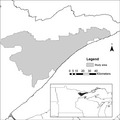

## 1 INTRODUCTION

Assessing ecology and management of wildlife requires knowledge of population trends over time. Surveys can reveal trends but their effectiveness to do so depends on their precision and accuracy (ArchMiller, Dorazio, St. Clair, & Fieberg, 2018). Examining trend data can be used to estimate a population's demographic rates, which can otherwise be costly and invasive to obtain through tracking of a representative sample of individuals. Conversely, rates of adult mortality and recruitment can be used to estimate population growth rates when and where surveys are not feasible, such as in densely forested regions or for cryptic species that occur at low densities (DeCesare et al., 2012; Hatter & Bergerud, 1991; Serrouya et al., 2017).

**Figure 1:**
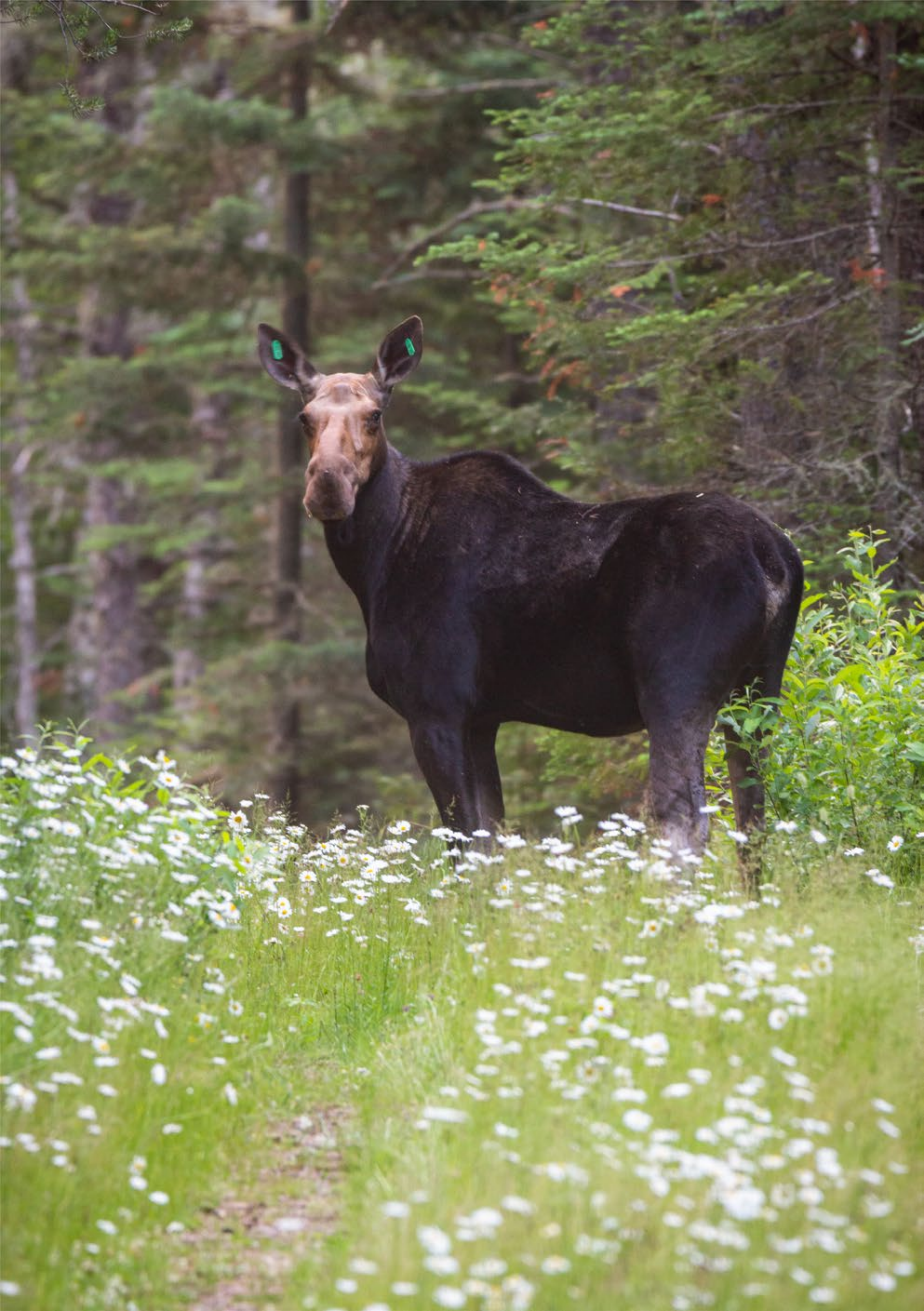
Adult moose (*Alces alces* Linnaeus, 1758) near Tofte, Minnesota. This moose was ear‐tagged as a neonate for a Minnesota Department of Natural Resources calf survival study and is about 5 years old in this image. Photo credit: Thomas Spence

By 2017, Minnesota's northeastern moose (*Alces alces* Linnaeus, 1758; Figure 1) population (3,710) was 58% lower than at its high point (8,840) in 2006, but it appeared to have stabilized during 2012–2017 (DelGiudice, 2017). A study of demographics of the northeastern population in 2002–2008 predicted a slow reduction in numbers (long‐term stochastic annual growth rate of 0.85); modeled adult survival rates were 0.74–0.85, and calf survival was 0.24–0.56 (Lenarz, Fieberg, Schrage, & Edwards, 2010). However, the abrupt decline in northeastern Minnesota was not detected by the annual aerial surveys until 2010 (ArchMiller et al., 2018; DelGiudice, 2013; Lenarz et al., 2010), which illustrated that demographic modeling may reveal population trajectories before they are reflected in total population estimates by aerial survey.

In response to the precipitous decline in the northeastern population, in 2012 the Minnesota Department of Natural Resources (MNDNR) initiated studies of adult and calf survival and cause‐specific mortality. These studies built upon previous research (Lenarz et al., 2010; Lenarz, Nelson, Schrage, & Edwards, 2009), but aimed to better understand causes of mortality (Butler, Carstensen, & DelGiudice, 2011; DelGiudice, Severud, Butler, Carstensen, & Moen, 2012). The more recent research employed state‐of‐the‐art global positioning system (GPS) collars and other remote monitoring techniques (e.g., internal temperature monitors, movement analyses) to track survival, habitat use, mortality, and physiological condition (Carstensen et al., 2018; DelGiudice, Severud, Obermoller, & St‐Louis, 2018; DelGiudice et al., 2015; Herberg, 2017; Obermoller, 2017; Severud, DelGiudice, & Obermoller, 2017; Severud, DelGiudice, Obermoller, Enright, et al., 2015).

Our goal was to compare estimates of population growth rate (*λ*) derived from demographic information from the adult moose and calf studies versus from the annual aerial surveys, because each source of data has inherent limitations (Hatter & Bergerud, 1991). Aerial surveys are relatively less costly compared to extensive collaring studies and can cover larger spatial extents. However, there is more precision and a greater detail of information gained from collaring studies. We projected population dynamics for 50 years using each method to gauge how current trends may affect the population's future. We examined the local sensitivity of all parameters to determine which data may be most important to predicting population growth. To model how variability in demographic rates may affect trajectories, we also employed a stochastic model to project the population for 30 years using adult survival rates and litter sizes.

### 1.1 Study area

**Figure 2:**
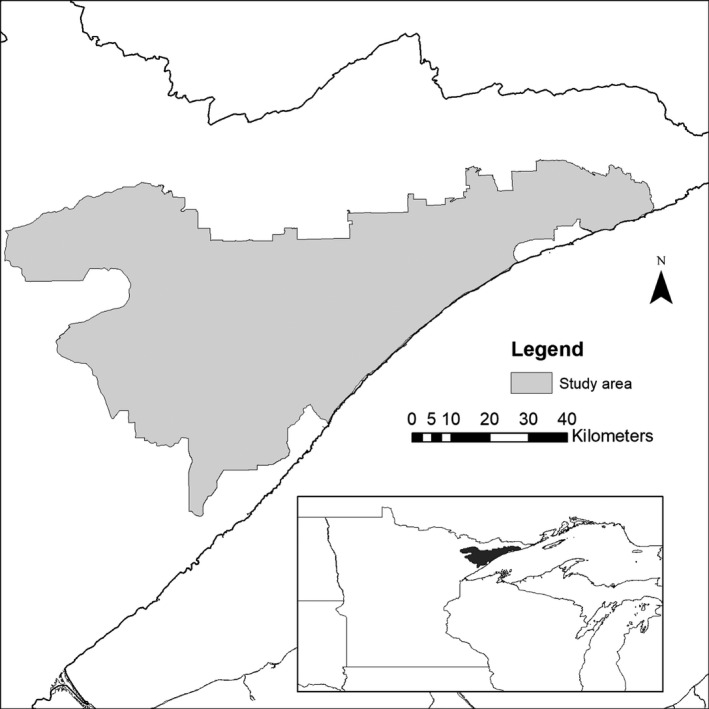
Study area of Minnesota Department of Natural Resources demographic moose studies (6,068 km^2^ study area) during May–June 2013–2016, northeastern Minnesota, USA. Annual aerial survey largely overlaps this study area

The MNDNR's demographic studies and aerial surveys were conducted in northeastern Minnesota along the edge of moose range in North America (Figure 2; Lenarz et al., 2010; Timmermann & Rodgers, 2017). This population of moose inhabits a mosaic of the Superior National Forest and various state, county, and private lands (6,068 km^2^) between 47°06′N and 47°58′N latitude and 90°04′W and 92°17′W longitude (Figure 2). Moose harvest was suspended in Minnesota from 2013 until 2016, when a limited tribal harvest was resumed (DelGiudice, 2012; Edwards, 2018; Schrage, 2018). This region is part of the Northern Superior Upland within the Laurentian mixed forest province (MNDNR, 2015). The vegetative cover is a mosaic of wetlands, stands of northern white cedar (*Thuja occidentalis*), black spruce (*Picea mariana*), and tamarack (*Larix laricina*), and upland stands of balsam fir (*Abies balsamea*), jack pine (*Pinus banksiana*), eastern white pine (*P. strobus*), and red pine (*P. resinosa*), intermixed with quaking aspen (*Populus tremuloides*) and paper birch (*Betula papyrifera*). Area of timber harvest and other forest disturbance declined 65% from 2001 to 2009 (Wilson & Ek, 2013).

Predators of moose calves were gray wolves (*Canis lupus* Linnaeus, 1758) and American black bears (*Ursus americanus* Pallas, 1780; Lenarz et al., 2009; Patterson et al., 2013; Severud, DelGiudice, Obermoller, Enright, et al., 2015; Severud, DelGiudice, Obermoller, Ryan, & Smith, 2015); wolf and bear densities were estimated at 4.4/100 and 23/100 km^2^, respectively (Garshelis & Noyce, 2011; Mech, Fieberg, & Barber‐Meyer, 2018). White‐tailed deer (*Odocoileus virginianus* Zimmermann, 1780), managed at prefawning densities of <4/km^2^, were primary prey of wolves in the area (DelGiudice, Riggs, Joly, & Pan, 2002; MNDNR, 2012; Nelson & Mech, 1981). Alternate wolf prey included adult moose, beavers (*Castor canadensis* Kuhl 1820), snowshoe hares (*Lepus americanus* Erxleben, 1777), black bears, and various small mammals (Chenaux‐Ibrahim, 2015; Frenzel, 1974; Stenlund, 1955; Van Ballenberghe, Erickson, & Byman, 1975).

## 2 METHODS

### 2.1 Aerial surveys

The MNDNR conducts an aerial survey of the northeastern moose population each winter (DelGiudice, 2019). The current survey design and methods were implemented in 2005. The survey area is ~15,500 km^2^ and is divided into 436 total survey plots, each ~36 km^2^. Each winter, 36–52 of the survey plots are chosen from a stratified random sample based on moose density (low, medium, high). The survey provides estimates of abundance (including 90% confidence intervals [CI]), percent calves, calf:cow and bull:cow ratios, and percent cows observed with twins. A sightability model corrects for visual obstruction and is used to adjust abundance (ArchMiller et al., 2018; Fieberg, 2012; Giudice, Fieberg, & Lenarz, 2012; Steinhorst & Samuel, 1989), but raw data, adjusted for sampling, are used to calculate other metrics using the combined ratio estimator (Cochran, 1977). The sightability model was based on radiocollared moose during 2004–2007 (Lenarz et al., 2010, 2009); logistic regression indicated visual obstruction was the most influential covariate in moose detection, so visual obstruction (proportion obstructed by vegetation) is estimated within a ~10‐m radius around the first observed moose in a group (Giudice et al., 2012). A linear trend line is fit to all data (2005 onward) each year, but a piecewise polynomial regression line has been fit beginning in 2016 to account for periods of stability and change (DelGiudice, 2016; Giudice, 2017). The piecewise regression used a spline function with 1 *df* and knots at 2009 and 2012 (Giudice, MNDNR, personal communication).

### 2.2 Adult and calf survival rates

Adult moose were captured by aerial darting and handled during winters 2013–2015 (Carstensen et al., 2018). Immobilizations were conducted with carfentanil, thiafentanil, and xylazine, and reversed with naltrexone and tolazoline (Carstensen, Hildebrand, Pauly, Wright, & Dexter, 2014). Moose were fitted with GPS‐Iridium collars (Vectronic Aerospace GmbH) variably programmed to collect a location every 4 hr during July–April, but every hour for females during calving (May–June).

Calves were monitored for survival during 2013–2016, but were fitted with GPS collars in 2013 and 2014 only (Obermoller, DelGiudice, & Severud, 2019; Severud, Obermoller, DelGiudice, & Fieberg, 2019). In 2013 and 2014, we monitored 74 neonates for survival (hourly fixes, Vectronic Aerospace GmbH). In 2015 and 2016, we monitored 50 and 35 calving females for signs of neonatal mortality using changes in adult female velocities and assessed seasonal calf survival by aerial surveys (Obermoller et al., 2019; Severud et al., 2019). Adult females were blood sampled to test for pregnancy; a threshold of ≥2 ng/ml of serum progesterone indicated pregnancy (Haigh, Kowal, Runge, & Wobeser, 1982; Murray et al., 2006; Testa & Adams, 1998). We estimated pregnancy rates using these test results and observations of cows that made a calving movement (Severud, DelGiudice, Obermoller, Enright, et al., 2015). Annual Kaplan–Meier survival rates were estimated for pooled adult (>1.5 years) males and females and for calves (birth to 1 year) during 2013–2016 (Carstensen et al., 2018; Obermoller, 2017; Severud et al., 2017).

### 2.3 Population growth rate calculation (*λ*)

We estimated *λ* using two different methods. First, to calculate *λ*_survey_ we used population estimates from the annual aerial survey and the equation:

$$\lambda_{\text{survey}}={\left(\frac{N_{t}}{N_{0}}\right)}^{\left(\frac{1}{t}\right)}$$

where *N* was the population estimate, and *t* is the time interval between surveys. *N*_0_ is the population at time 0.

Second, we used the recruitment–mortality (R‐M) Equation (Hatter & Bergerud, 1991) to calculate *λ*_R‐M_:

$$\lambda_{R\;-\;M}=\frac{\left(1-M\right)}{\left(1-R\right)}$$

where *M* is the finite annual adult mortality rate, and *R* is the finite annual recruitment rate defined as the calf proportion of the population. We used published adult survival estimates from MNDNR's adult collaring study (*S*_adult_) to calculate mortality using 1 – *S* = *M* (Carstensen et al., 2018). To obtain estimates of *R*, we used the population estimate, bull:cow ratio (DelGiudice, 2017), mean twinning rate (M. W. Schrage, Fond du Lac Resource Management Division, unpublished data), pregnancy rates, and annual calf survival from GPS‐collared and uncollared calves (Obermoller et al., 2017; Severud et al., 2017). We used estimates from the previous year to calculate the current year's *R* (e.g., 2013 adult population estimate [total population estimate minus calf proportion], bull:cow ratio [from which we derived proportion cows], pregnancy rate, and calf survival to calculate 2014's *R*), because moose are considered recruited once they reach 1 year of age. First, we calculated calf production as:

$$\begin{array}{c} \text{calf}\;\text{production}=\left(\text{adult}\;{\text{pop}}^{\prime }n\;\text{estimate}\times \text{proportion}\;\text{cows}\times \text{preg}\;\text{rate}\right)\\ +\;\left(\text{adult}\;{\text{pop}}^{\prime }n\;\text{estimate}\times \text{proportion}\;\text{cows}\times \text{preg}\;\text{rate}\times \text{twinning}\;\text{rate}\right) \end{array}$$

We then used calf survival to calculate *R*_study_ as:

$$R_{\text{study}\;t}=\frac{\text{calf}\;{\text{survival}}_{t-1}\times \text{calf}\;{\text{production}}_{t-1}}{{\text{pop}}^{\prime }n\;{\text{estimate}}_{t-1}}$$

*M* can also be calculated by rearranging the R‐M Equation to:

$$M=1-\lambda \left(1-R\right)$$

Using this equation, we estimated adult mortality rates from *λ*_survey_ and *R*_survey_ (percent calves as reported in the survey) to compare how closely they tracked mortality rates and percent calves as calculated from the demographic study (i.e., *R*_study_ vs. *R*_survey_ and *λ*_R‐M_ vs. *λ*_survey_).

### 2.4 Population projection

We calculated median and standard deviation of *S*_adult_ and calf:cow ratios at calving (litter size). We then used the 2014 population estimate for the initial population (4,350 adults; used to coincide with results of collaring study) and projected growth for 30 years and 1,000 Monte Carlo simulations using the R package *population* (Chapron, 2015). We also projected the population for 50 years using mean *λ*_survey_ from the recent stable period (2012–2017) and from the entire period (2005–2017), mean *λ*_R‐M_ (2013–2016, duration of demographic studies), and the long‐term stochastic growth rate from a previous study (0.85; Lenarz et al., 2010; see above). We projected populations 50 years to match climate scenario and long‐term forest planning timeframes. We investigated local sensitivity of all parameters used to calculate *λ*_R‐M_ by incrementally increasing a single parameter while holding the others at mean levels until *λ* increased from 1.00 to 1.10, a level which would reverse the population decline (Hamby, 1994; MNDNR, 2012; Serrouya et al., 2017).

## 3 RESULTS

The MNDNR collared 173 adult moose from 2013 to 2015 (123 F, 50 M) to assess survival and cause‐specific mortality (Carstensen et al., 2018). Survival was pooled for males and females due to a small sample of male mortalities. Adult annual survival estimates for 2013–2016 were 0.81, 0.88, 0.86, and 0.85, respectively (Carstensen et al., 2018). Seventy‐four neonates were collared in 2013 and 2014 (combined), with an additional 103 uncollared calves of GPS‐collared cows monitored for survival in 2015 (*n* = 65) and 2016 (*n* = 38). Estimated annual calf survival rates were 0.28, 0.40, 0.40, and 0.33, for 2013–2016, respectively (Obermoller et al., 2017; Severud et al., 2017).

**Figure 3:**
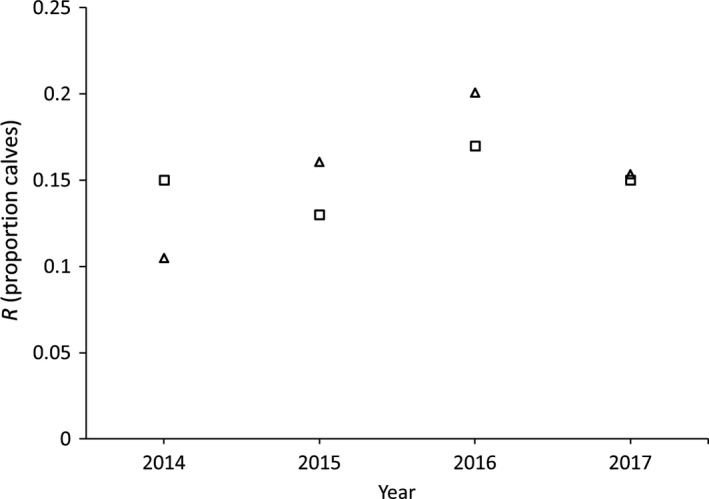
Estimated moose (*Alces alces*) recruitment (*R*; calf proportion of the total population) observed during the annual aerial survey (squares; *R*_survey_; DelGiudice, 2017) and calculated using parameters from a calf survival study (triangles; *R*_study_; Obermoller et al., 2017; Severud et al., 2017), 2014–2017, northeastern Minnesota

The annual aerial survey reported population estimate, calf:cow ratio, percent of the population composed of calves, percent of cows observed with twins, and bull:cow ratio (DelGiudice, 2017). We estimated pregnancy rates of 0.74, 0.81, 0.88, and 0.89 for 2013–2016, respectively. We used a mean twinning rate of 30% (M. W. Schrage, Fond du Lac Resource Management Division, unpublished data). Mean *R*_study_ was 0.15 (range = 0.10–0.20), whereas mean *R*_survey_ for the same time period was also 0.15 (range = 0.13–0.17; Table 1). *R*_survey_ and *R*_study_ closely tracked each other for all years (2014–2017; Figure 3).

**Table 1:** Demographic parameters of moose (*Alces alces*) derived from annual aerial surveys and studies of adult and calf survival, 2013−2017, northeastern Minnesota

Survey year	*N* a	*M*_adult_ b	*S*_calf_ c	*R*_survey_ a	*R*_study_ c	Preg rate	Calf production	*λ*_survey_	*λ*_R‐M_
2013	2,760	0.19	0.28	–	–	0.74	1,040	0.65	–
2014	4,350	0.12	0.40	0.15	0.10	0.81	1,747	1.58	0.90
2015	3,450	0.14	0.40	0.13	0.16	0.88	1,732	0.79	1.05
2016	4,020	0.15	0.33	0.17	0.20	0.89	1,893	1.17	1.08
2017	3,710	–	–	0.15	0.15	0.85	1,824	0.92	1.00
Mean		0.15	0.35	0.15	0.15	0.83	1,647	0.99	1.01
*SE*		0.01	0.03	0.01	0.02	0.03	154	0.16	0.04

Note

*N* is the population estimate, *M* is the annual adult mortality rate, *S* is calf survival, *R* is recruitment (calf proportion of the population), and preg rate is pregnancy rate as determined by serum progesterone, calving behavior, and calf observations. *λ*_survey_ was calculated using changes in population estimates; *λ*_R‐M_ was calculated using the R‐M Equation, (1 − *M*)/(1 − *R*). No data or data from outside our time‐frame are indicated by (–).

a DelGiudice (2017).

b Carstensen et al. (2017) and M. Carstensen (MNDNR, personal communication).

c Obermoller et al. (2017) and Severud et al. (2017).

**Figure 4:**
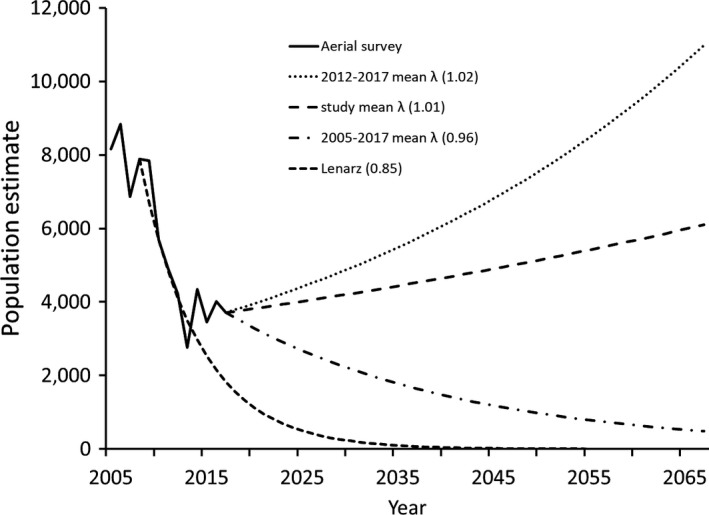
Population estimates of moose (*Alces alces*) in northeastern Minnesota (2005–2017) with deterministic projections using 3 calculations of *λ* from 2017 onward (*λ*_R‐M_, *λ*_survey_ for 2012–2017, and *λ*_survey_ for 2005–2017), and one projection from 2008 onward (*λ* = 0.85 from Lenarz et al., 2010)

We estimated mean *λ*_survey_ for three different intervals based on a piecewise polynomial which indicated the population had gone through three distinct growth phases: stability (2005–2009), decline (2009–2012), and then stability again (2012–2017; DelGiudice, 2017). The initial stable phase *λ*_survey_ was 1.00, followed by 0.82 during the decline, and 1.02 during the most recent stable phase. Extreme values of *λ*_survey_ were influenced by the notably low, and likely unrealistic (see Section 4) population estimate of 2013. The highest *λ*_survey_ for a single year was 2014 (1.58), when the population estimate increased dramatically following the low population estimate of 2013 (Figure 4). Similarly, the lowest estimate of *λ*_survey_ was in 2013 (0.65) when the population dropped markedly (Table 1).

**Figure 5:**
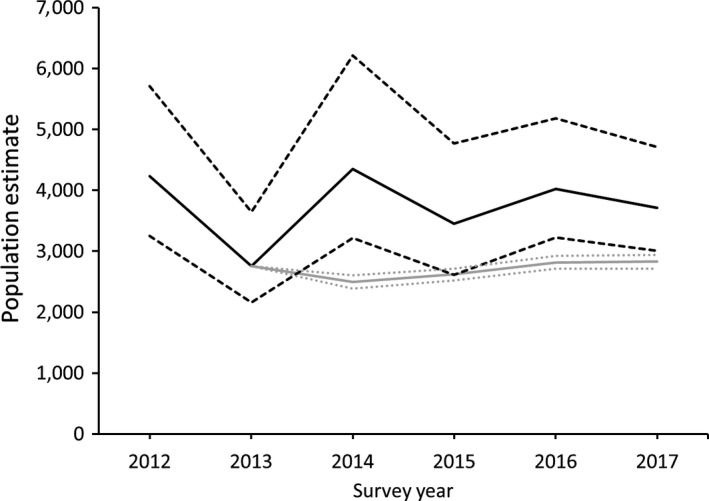
Population estimate of moose (*Alces alces*) in northeastern Minnesota (solid black line) plus 90% confidence intervals (CI; dashed black lines) during the 2012–2017 apparent stabilization (DelGiudice, 2017), and modeled population (solid gray line) plus 90% CI (dotted gray lines) using *λ*_R‐M_ from 2013 onward

Mean *λ*_R‐M_ for 2014–2017 was 1.01 (±0.04 [*SE*], range = 0.90–1.08, *n* = 4), indicative of a slightly growing population, and similar to estimates of *λ*_survey_ during the recent stabilization (1.02 ± 0.16; 2012–2017). The highest observed *λ*_R‐M_ was 1.08 for 2016 (Table 1), when both the previous year's adult mortality were relatively low (14%) and calf survival was high (40%). The previous year's pregnancy rate was also high (0.88). Using the 2013 population estimate as a common starting point, we used *λ*_R‐M_ to predict annual population for 2014–2017 (Figure 5). The modeled projection was relatively stable and generally slightly lower than the lower 90% confidence intervals of the observed survey estimates. Adult mortality rates (*M*_adult_) widely varied, but the mean during 2013–2017 was 13.0% which is similar to the adult study average of 13.8%. However, the R‐M Equation calculated a negative mortality rate in 2014 (−34%).

Holding all other parameters at mean rates (*S*_adult_ = 0.85, twinning rate = 0.30, pregnancy rate = 0.83, proportion cows = 0.48, mean population = 5,593), an increase of *S*_calf_ from 0.285 to 0.435 (0.15 difference) increased *λ*_R‐M_ from 1.00 to 1.10. However, if *S*_calf_ is held constant at 0.285, an increase from 0.850 to 0.935 (0.085 difference) in *S*_adult_ results in the same change in *λ*. A similar increase of *λ* from 1.00 to 1.10 would require the bull:cow ratio to change to 75% cows or increasing twinning rate to 100%. A 100% pregnancy rate would only increase *λ* from 1.00 to 1.03.

**Figure 6:**
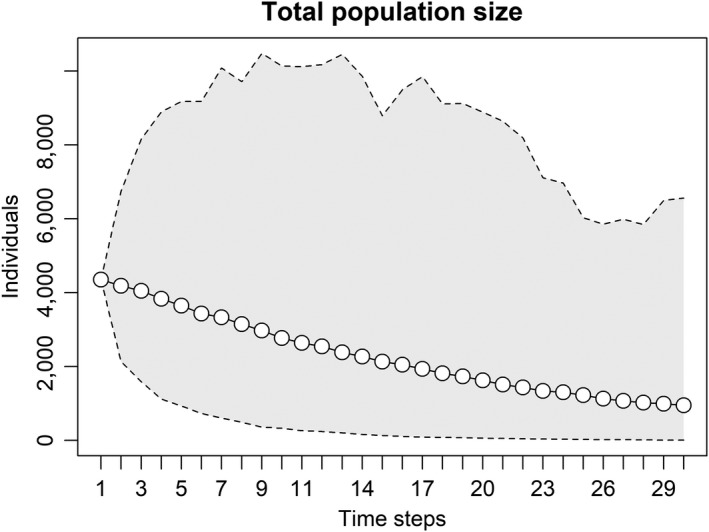
Stochastic population projection (30 years) of moose (*Alces alces*) in northeastern Minnesota from 2014 (*N* = 4,350) onward using median ± standard deviation of adult survival (0.85 ± 0.04) and litter size (calf:cow ratio at birth, 1.12 ± 0.32). Shaded area represents limits from 1,000 Monte Carlo simulations using the R package *population*

Deterministic projections using mean *λ*_survey_ from the recent stable period (2012–2017; 1.02) resulted in a growing population (Figure 4). Using *λ*_survey_ from 2005–2017 (0.96) resulted in a slowly declining population. Mean *λ*_R‐M_ (1.01) resulted in a slightly increasing population over 50 years. The matrix projection from Lenarz et al. (2010) (0.85 from 2002 to 2008) indicated a declining population, yet survey results have been above the trajectory for 2014–2019. The stochastic model using median demographics (adult survival = 0.85 ± 0.03 [*SD*], calf:cow ratio at birth = 1.12 ± 0.32) from the MNDNR studies shows the population declining, but with uncertainty (*λ* = 0.91 ± 0.04; Figure 6).

## 4 DISCUSSION

We projected the northeastern Minnesota moose population using three calculations of *λ* (*λ*_R‐M_, *λ*_survey_ for 2012–2017, and *λ*_survey_ for 2005–2017) and one from the literature (Lenarz et al., 2010). Two of these projections predicted an increasing population and 2 indicated a decreasing trend. The increasing trajectories used more recent estimates of *λ* (2012–2017 survey‐derived [*λ*_survey_ for 2012–2017] and 2014–2017 study‐derived [*λ*_R‐M_]). The stochastic projection resulted in a steadily decreasing population. These estimates of *λ* are reflective of more current conditions (e.g., environmental, demographic, harvest pressure), which may change through time and increase standard errors in predictions (Ellner & Fieberg, 2003). Comparing deterministic and stochastic projections can yield opposing results (Nakaoka, 1996). Using stochastic projections may be more appropriate in cases with greater environmental variability (Boyce, 1977; May, 1973).

Climate change, specifically warming temperatures, is expected to influence moose demographics at the southern periphery of their geographic range (Lenarz et al., 2010; Lenarz et al., 2009; McCann, Moen, & Harris, 2013; Murray et al., 2006; Ruprecht et al., 2016; but see Mech & Fieberg, 2014). The northeastern Minnesota moose population has shown some response to warmer than average winter temperatures, including reduced survival (Lenarz et al., 2009; but see Mech & Fieberg, 2014) and an increase in winter nutritional restriction (DelGiudice & Severud, 2017). Similarly, behavioral changes (e.g., use of habitat) in response to warm temperatures have been observed (McCann, Moen, Windels, & Harris, 2016; Street et al., 2016; Ditmer et al., 2018). The explicit effect of higher temperature on moose survival has yet to be fully addressed.

Annual estimates of *λ* calculated from survey data (*λ*_survey_) varied widely from 0.65 to 1.58, whereas *λ* calculated using the R‐M Equation from the adult and calf demographic studies (*λ*_R‐M_) ranged from 0.90 to 1.08. The extreme values of *λ* derived from the survey (*λ*_survey_) are both strongly influenced by the low 2013 population estimate. This point estimate was associated with poor survey conditions and, consequently, has been considered an outlier (G. D. DelGiudice, MNDNR, personal communication). Our modeled projection using 2013 as a starting point (Figure 5) also suggests that the actual population in 2013 was likely closer to the upper 90% confidence interval. The large variation in *λ*_survey_ also underscores that the survey results should be used to assess long‐term trends rather than year‐to‐year changes in the population.

Growth rates calculated from the adult and calf studies suggest that the northeastern Minnesota moose population is slowly growing (about 1% per year). The *λ*_survey_ associated with the stability of 2012–2017 is similar to estimates from the R‐M Equation (1.02 vs. 1.01). Although the point estimates of the annual survey do not yet appear to reflect an increase in total population, there could potentially be a time lag before the upward trend is apparent. The 2018 and 2019 estimates (3,030 and 4,180, respectively) continue to indicate a stable population (i.e., within 90% CI; DelGiudice, 2018, 2019). Previous research in northeastern Minnesota reported declining population projections despite the annual survey not yet revealing the decline (Lenarz et al., 2010), suggesting that a large increase in the population may be required before a significant change in the survey's point estimates is observed versus an increasing trend.

Varying adult survival had more of an impact on *λ* than varying calf survival or any other parameter contributing to *R* in the R‐M Equation (twinning rate, pregnancy rate, bull:cow ratio). Previous research similarly concluded that fertility, calf survival, and adult survival explained 5%, 11%, and 70% of the variation in *λ*, respectively (Lenarz et al., 2010). The feasibility of applying management strategies and activities that sufficiently alter bull:cow ratios, or increase twinning or pregnancy rates to 100% necessary to markedly affect *λ* is unlikely. The population is already near its maximum reproductive output.

Survival of adult large herbivores is generally high and more consistent than juvenile survival (Gaillard, Festa‐Bianchet, & Yoccoz, 1998; Gaillard, Festa‐Bianchet, Yoccoz, Loison, & Toigo, 2000), so low and highly variable calf survival can greatly impact population dynamics (Lenarz et al., 2010; Raithel, Kauffman, & Pletscher, 2007; Serrouya et al., 2017). The MNDNR demographic studies showed adult survival rates were three times more stable than calf survival (*SE*s of 0.01 vs. 0.03). Calf survival in our study ranged from 0.28 to 0.40; survival consistently nearer 0.40 could have promoted population growth (i.e., *λ* > 1, based on sensitivity analyses).

Our calculations of *R*_study_ closely tracked *R*_survey_, but because there are assumptions (e.g., twinning rate) and uncertainty (calf survival) surrounding parameters used to calculate *R*_study_, the close association should be interpreted cautiously. In April 2015, collaring moose was banned and new methods were developed to monitor calf survival (Obermoller et al., 2019; Severud et al., 2019). Being newly developed and implemented, the 2015 survival estimate warrants prudent interpretation, yet closely aligns with survival estimates in the years before and after 2015. Furthermore, methods used to estimate recruitment in all years of the calf survival study and from the aerial survey (conducted in January) may have missed late‐winter mortality observed elsewhere (Jones, Pekins, Kantar, O'Neil, & Ellingwood, 2017; Musante, Pekins, & Scarpitti, 2010; Serrouya et al., 2017).

To estimate population demographics, a large and geographically dispersed sample of that population is followed to ensure the sample is representative. The MNDNR collared large numbers of adult moose and calves across the study area. The goal of the adult survival study was to maintain an annual starting sample of about 100 animals transmitting throughout the duration of the study; however, the indefinite moratorium on collaring moose plus attrition of existing collars due to mortalities, battery‐life expiration, and malfunction greatly reduced sample size to less than half by 2016 (Carstensen et al., 2017). The adult mortality rate has generally been decreasing, but later estimates could be biased due to weakened animals being culled from the population through predation or health‐related mortality and stronger animals surviving to later years of the study. A relationship between collared adult survival rates and population‐wide assessments of winter nutritional restriction suggests the condition of the collared animals was representative of that of the free‐ranging population in the earliest years of the study (DelGiudice & Severud, 2017). If collaring demographic studies can no longer be conducted in Minnesota, *λ*, *R*, and *M* can be estimated via the annual aerial survey; however, *λ* and *M* can be influenced by wide fluctuations in the population point estimate, as was seen before and after 2013. After the large increase from 2013 to 2014, an artifact of survey condition in 2013, a negative mortality rate was observed, as was seen in other rapidly increasing populations (Hatter & Bergerud, 1991). An integrated population model using survey estimates combined with demographic data from the MNDNR studies may offer another approach to better understand trends in the population (Besbeas, Freeman, Morgan, & Catchpole, 2002). Reliance solely upon the survey to understand moose population dynamics will not be as informative or useful in the absence of demographic data gained from collaring studies, as has been demonstrated for fisher (*Pekania pennanti* Erxleben 1777) and caribou (*Rangifer tarandus* Linneaus 1758; Berg, Erb, Fieberg, & Forester, 2017; Murray et al., 2006; Serrouya et al., 2017). Collared animals are also needed to periodically recalibrate sightability models (Serrouya et al., 2017). Thus, the biologically significant value of resumed collaring cannot be overstated.

## 5 CONCLUSIONS

Aerial survey results indicate that the moose population was stable during 2012–2017, and population modeling suggests that the population may have increased in the short‐term; however, over the long‐term, models made varying predictions about the direction of the population trajectory. With collaring no longer possible to track the population, we can still estimate *R* and *M* from the survey. The parameter *R* integrates fecundity and calf survival, but cannot indicate timing and cause of mortality or twinning rates, all of which are of keen interest and value to management to understand population dynamics. Estimates of *M* can be unrealistic given the low precision of the population estimate. The R‐M Equation has utility, but supportive information from demographic collaring studies is critical to further address management questions.

## CONFLICT OF INTEREST

None declared.

## AUTHOR CONTRIBUTIONS

WJS and GDD conceived the initial ideas and collected data; WJS primarily conducted data analysis with help from GDD and JKB; WJS, GDD, and JKB contributed critically to writing the manuscript and gave final approval for publication.

## Data Availability

Data are available at the Data Repository for the University of Minnesota (DRUM), https://doi.org/10.13020/bd01-3547.
